# Analysis of Ligation Activity in Miniaturized L1 Ligase Ribozyme Variants Down to 49 nt and in Split Ribozyme Forms

**DOI:** 10.3390/ijms27209107

**Published:** 2026-10-14

**Authors:** Yuki Nitta, Misaki Kato, Yunoka Takagi, Yusuke Saga, Tadashi Ando, Hiromi Mutsuro-Aoki, Koji Tamura

**Affiliations:** 1 Department of Biological Science and Technology, Tokyo University of Science, 6-3-1 Niijuku, Katsushika-ku, Tokyo 125-8585, Japan; yuki.8321541@gmail.com (Y.N.); kt.273y@gmail.com (M.K.); yunokatakagi@gmail.com (Y.T.); y.saga@rs.tus.ac.jp (Y.S.); haoki0930@gmail.com (H.M.-A.); 2 Department of Applied Electronics, Tokyo University of Science, 6-3-1 Niijuku, Katsushika-ku, Tokyo 125-8585, Japan; tando@rs.tus.ac.jp; 3 Research Institute for Science and Technology, Tokyo University of Science, 2641 Yamazaki, Noda 278-8510, Japan

**Keywords:** L1 ligase ribozyme, RNA, RNA world, origin of life, base pair

## Abstract

Identifying the smallest active unit capable of ligating RNA in the absence of proteins is important to elucidate ribozyme evolution. Here, we aimed to construct an evolutionary model of the L1 ligase ribozyme. The miniaturized L1 ligase ribozyme (L1X6c2) consists of three stems: Stems A, B, and C. Mutants with shortened stems B and C were used to examine whether ligation occurred. In the mutant where both stems were simultaneously shortened, no ligation products were observed. We also constructed mutants with nucleotide deletions, measured their ligation-product signals after 24 h, and identified the structures crucial for ligation. A split mutant, formed by cleaving L1X6c2 at stem B, showed a ligation-product signal after 24 h close to that of L1X6c2, whereas another split mutant, formed by cleaving L1X6c2 at stem C, exhibited decreased activity. Although non-Watson–Crick base pairs near the ligation site did not affect ligation, the presence of G1 was important for ligation. Mutational analysis suggested that strong interactions between G1 and A51 in the G1–A51–U38 base triple are responsible for the base specificity of A51. Moreover, consecutive base pairs on each stem subtly contribute to the stability of the ribozyme folded into a compact structure to create a catalytic pocket.

## 1. Introduction

In the unidirectional flow of genetic information known as the “central dogma” [1], DNA serves as the source of information for protein synthesis, whereas proteins are necessary for DNA transcription and RNA translation. However, the evolution of genetic information transfer on early Earth makes it difficult to believe that complex systems such as DNA, RNA, and proteins found in modern life forms appeared suddenly. This raises the question of which originated first on primordial Earth, DNA or proteins. Since the 1970s, the discoveries of reverse transcriptase [2] and ribozymes [3,4] have suggested that RNA not only stores genetic information similar to DNA but also exhibits enzyme activity similar to proteins. These discoveries led to the proposal of the RNA World Hypothesis [5].

**Figure 1:**
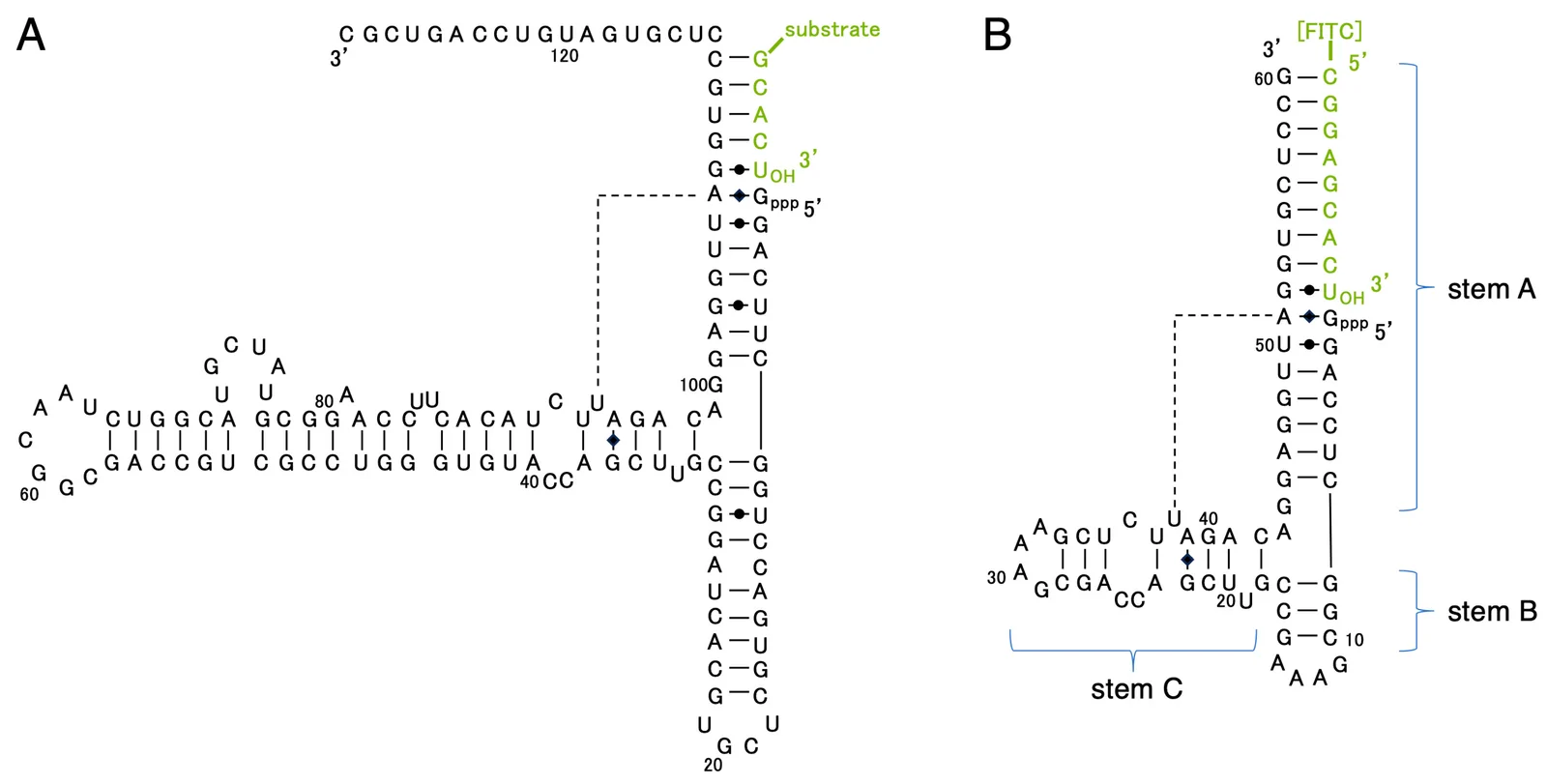
(**A**) Secondary structure of the full-length L1 ligase ribozyme (black) with an RNA substrate (green). The ribozyme is composed of 130 nucleotides. The dotted line indicates a base triple. (**B**) Secondary structure of the minimized construct L1X6c2 used in this study (black) with a fluorescently labeled RNA substrate (green). L1X6c2 is composed of 60 nucleotides. The dotted line indicates a base triple.

The linking of small RNA molecules is necessary to form larger structures. Several ligase ribozymes have been isolated via in vitro selection [6,7,8,9]. Typical examples include the L1 (130 nt; Figure 1A) [10,11,12], class I (121 nt) [13,14,15], R3C (73 nt) [16,17,18], and DSL (115 nt) [19] ligase ribozymes. The L1 [11] and R3C [20,21,22] ribozymes have been minimized, and L1 [11] and class I [23] ribozymes have been crystallized, with their three-dimensional structures determined. We previously reported that the class I ribozyme can still undergo ligation even after partial removal of the structure and splitting [24]. During evolution, a short RNA was possibly fused with a domain that interacted with a specific compound (effector), and the activity was regulated by and dependent on that specific effector [25,26].

The L1 ligase ribozyme (Figure 1A) catalyzes nucleophilic attack by a 3′-hydroxyl group on the α-phosphorus of its 5′-triphosphate, resulting the regiospecific formation of a natural 5′–3′ phosphodiester bond [8]. Previous efforts to delineate the sequence requirements for L1 ligase activity employed systematic deletion and optimization. Ellington and colleagues showed that the ribozyme could be substantially shortened while still undergoing ligation, identifying a 56-nt construct as a highly truncated, active form of the L1 ligase [27]. However, the extent to which L1 ligase activity could be retained upon further shortening or division of the ribozyme into separate RNA fragments remained unclear. In this study, we used the minimized L1 ligase ribozyme L1X6c2 (60 nt; Figure 1B), derived from the minimized crystallization construct L1X6c2 (71 nt) [11], and examined further shortened and split L1 ligase variants and found that ligation was still observed in a 49-nt variant as well as in a split form. These results provide further definition of the sequence and structural requirements for L1 ligase activity.

## 2. Results

**Figure 2:**
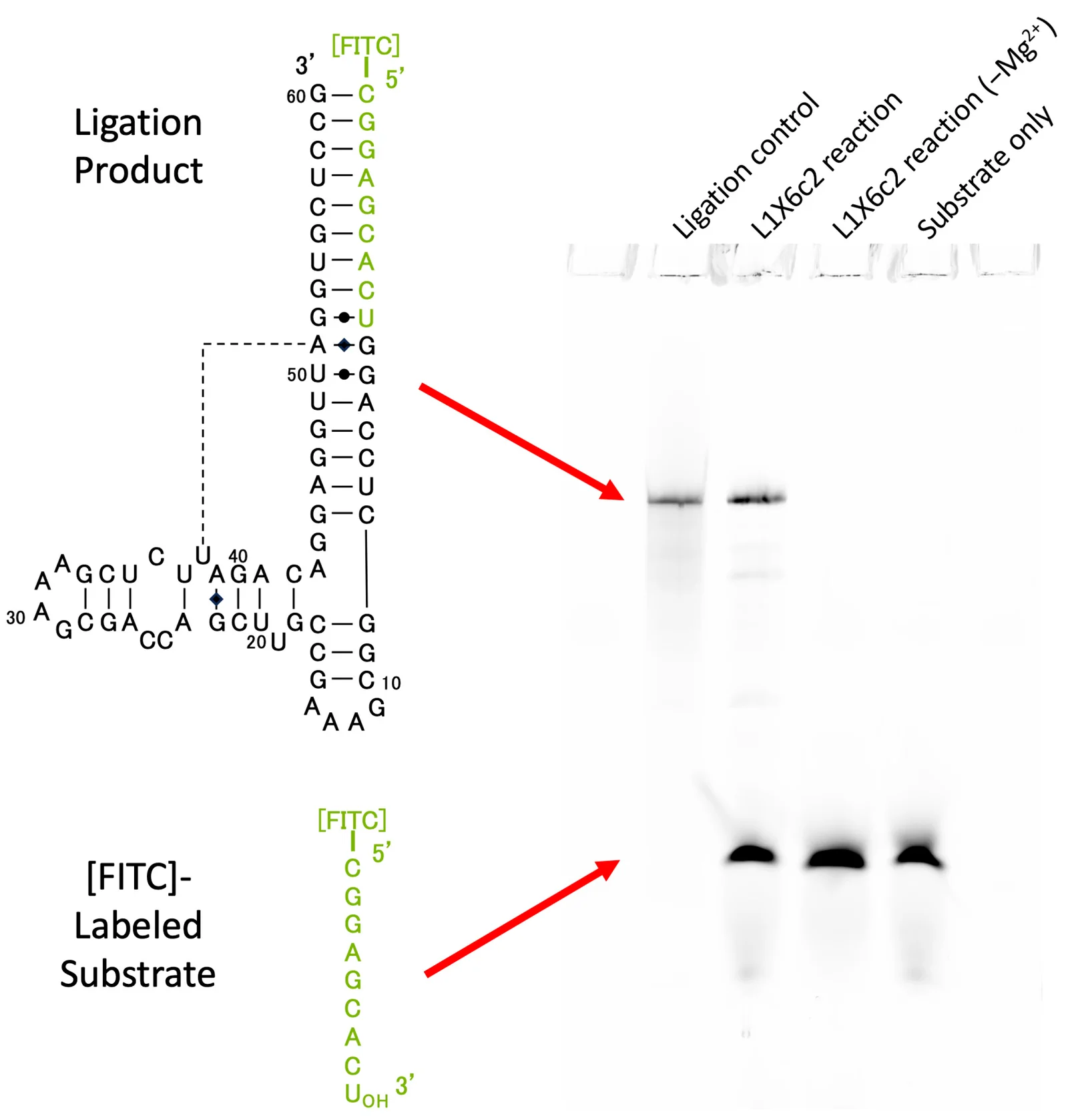
Mg^2+^-dependent ligation of L1X6c2. Ligation-product signal of L1X6c2 was analyzed by electrophoresis on a denaturing 12% polyacrylamide gel containing 7 M urea. The gel was visualized using a Typhoon FLA 7000 (GE Healthcare Japan, Tokyo, Japan). A fluorescently labeled 69-nt ligation-product was used as a size reference to identify the expected position of the ligation-product. Ligation control, L1X6c2 reaction, L1X6c2 reaction (−Mg^2+^), and substrate-only samples are shown.

L1X6c2 underwent ligation only in the presence of Mg^2+^. The migration position of the ligation product was confirmed by comparison with a fluorescently labeled ligation product (69 nt) of the expected length generated using L1X6c2 (Figure 2).

**Figure 3:**
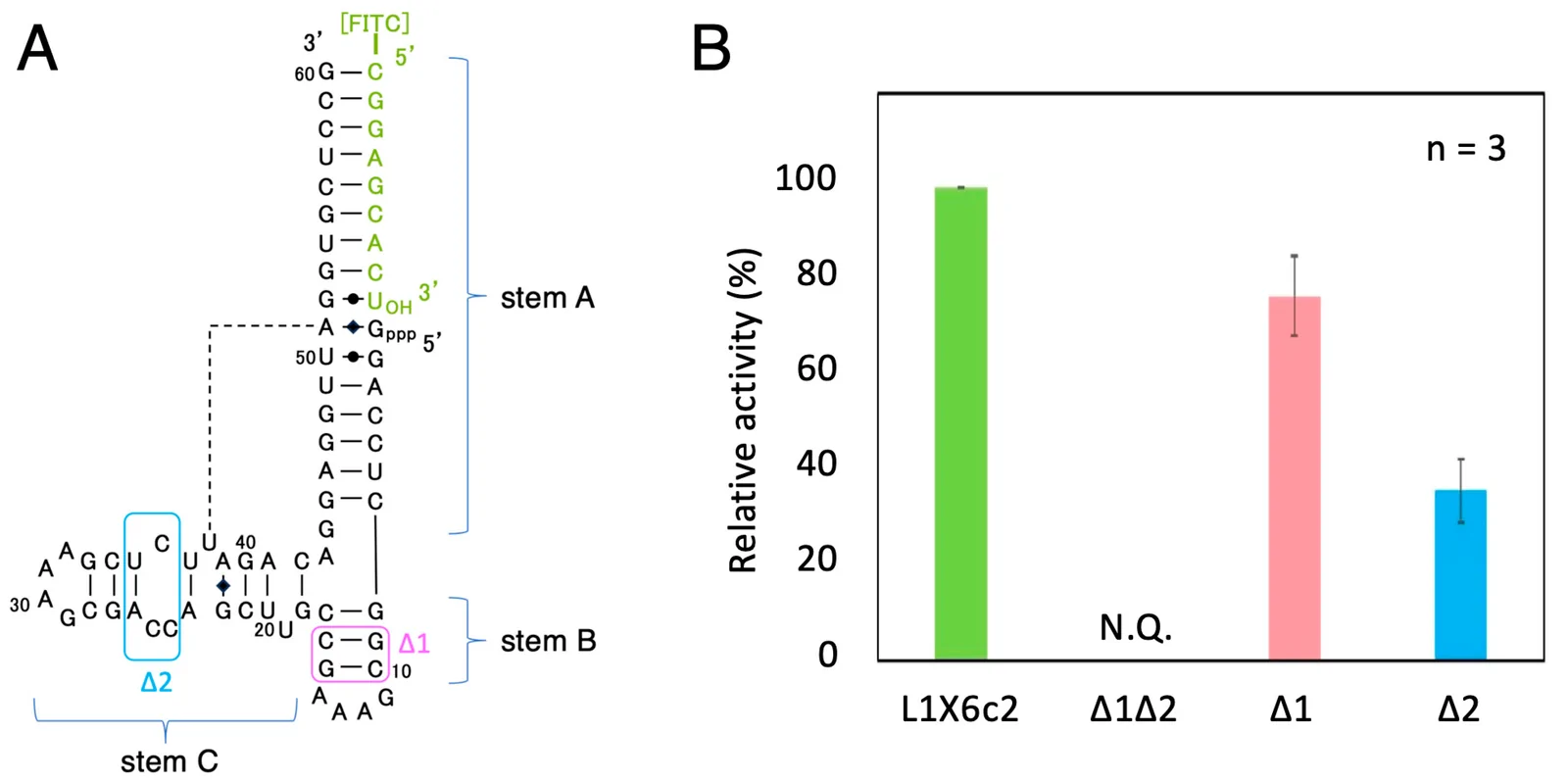
(**A**) Deletion mutants of the L1X6c2 ligase ribozyme. The regions enclosed by rounded rectangles (Δ1 and Δ2) were deleted to generate deletion mutants. The RNA sequences are also shown in the Supplementary Materials (Table S1). (**B**) Relative ligation-product signals after 24 h of the L1X6c2 ligase ribozyme and its deletion mutants. The gel was visualized using a Typhoon FLA 7000 (GE Healthcare Japan, Tokyo, Japan) and the ligation product band was quantified from the gel image using ImageQuant TL software, as described in the Section 4. The image of the gel is presented in the Supplementary Materials (Figure S1). Similar electrophoretic analyses and quantification were performed for the other experiments. The ligation-product signals are shown as relative values (%) compared to that of the L1X6c2 ligase ribozyme (100%). A signal was classified as N.Q. when the corresponding band could not be reliably quantifiable in any of the three independent PAGE analyses. Data are presented as mean ± SD from three independent experiments (*n* = 3).

To reduce the size of L1X6c2 while minimizing potential effects on its function, we initially selected the Δ1 and Δ2 regions for deletion, guided by the predicted secondary structure of L1X6c2 (Figure 3A). We generated the mutants Δ1, Δ2, and Δ1Δ2 by deleting the regions shown in Figure 3A from the L1X6c2 ribozyme. Δ1Δ2, which lacked some nucleotides in both stems B and C, did not show any reliably quantifiable ligation (Figure 3B). In contrast, Δ1 and Δ2, which lacked some nucleotides in stems B and C, respectively, still underwent ligation (Figure 3B). However, their relative ligation-product signals after 24 h were decreased by approximately 77 and 36%, respectively, compared to that of L1X6c2 (Figure 3B).

**Figure 4:**
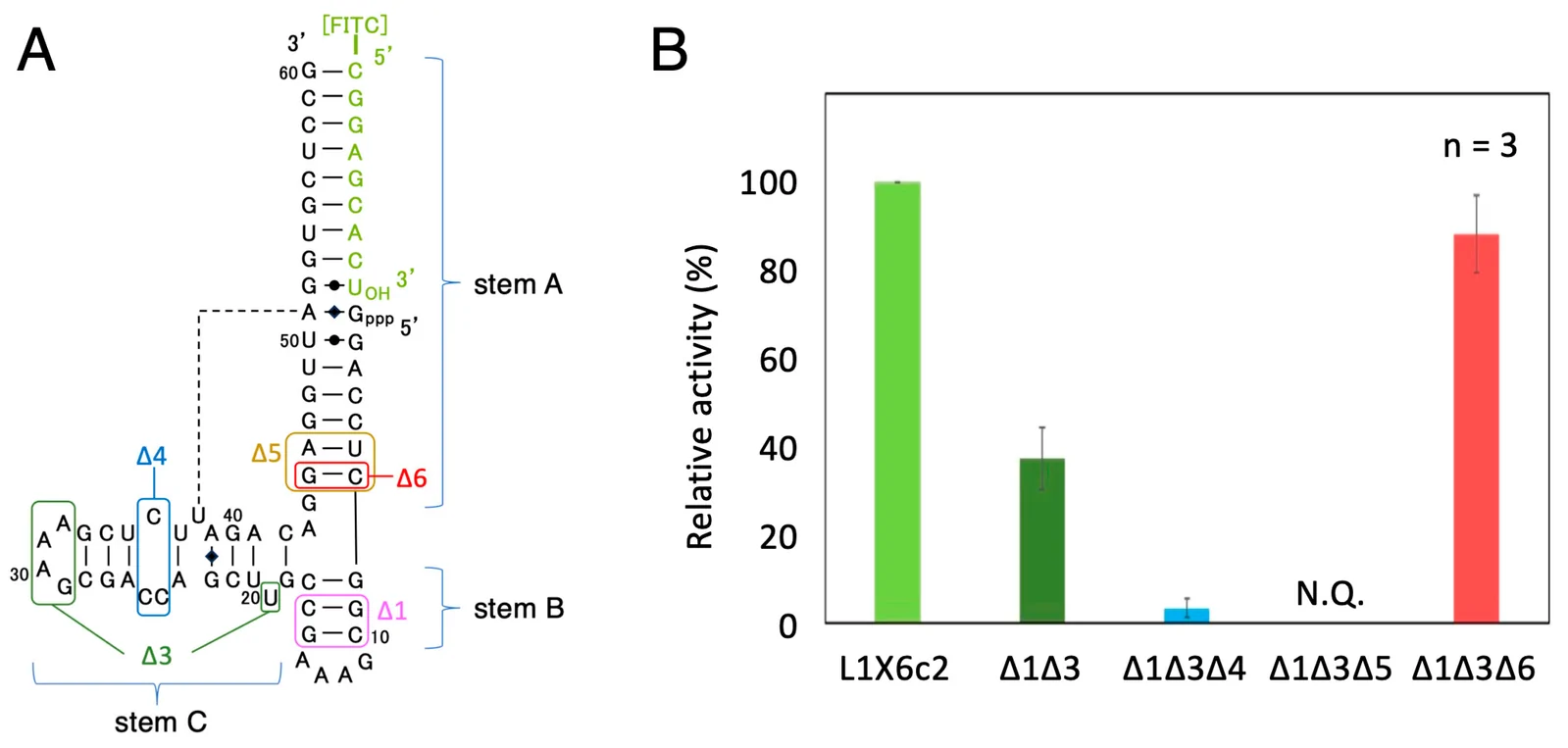
(**A**) Deletion mutants of the L1X6c2 ligase ribozyme. The regions enclosed by rounded rectangles (Δ1, Δ3, Δ4, Δ5 and Δ6) were deleted to generate deletion mutants. The RNA sequences were also shown in the Supplementary Materials (Table S1). (**B**) Relative ligation-product signals after 24 h of the L1X6c2 ligase ribozyme and its deletion mutants. The signals are shown as relative values (%) compared to that of the L1X6c2 ligase ribozyme (100%). A signal was classified as N.Q. when the corresponding band could not be reliably quantifiable in any of the three independent PAGE analyses. Data are presented as mean ± SD from three independent experiments (*n* = 3).

Based on these results, we next explored additional deletion sites to further reduce the size of L1X6c2 while still undergoing ligation. We generated the deletion mutant Δ1Δ3 (51 nt), in which nine nucleotides were deleted from L1X6c2 (Figure 4A). Its relative ligation-product signal after 24 h was approximately 37% of that of L1X6c2 (Figure 4B). Additionally, we constructed the deletion mutants Δ1Δ3Δ4 (deleting C24, C25, and C36, which protrude from Δ1Δ3 in the secondary structure, in Δ1Δ3; Figure 4A), Δ1Δ3Δ5 (deleting U6, C7, G45, and A46 in Δ1Δ3; Figure 4A), and Δ1Δ3Δ6 (deleting A46 and C7 in Δ1Δ3; Figure 4A) and compared their relative ligation-product signals after 24 h (Figure 4B). Although the relative ligation-product signals after 24 h of Δ1Δ3Δ4 and Δ1Δ3Δ5 were decreased, Δ1Δ3Δ6 (49 nt) showed a higher signal than Δ1Δ3 (51 nt), which was 88% of that of L1X6c2 (Figure 4B).

**Figure 5:**
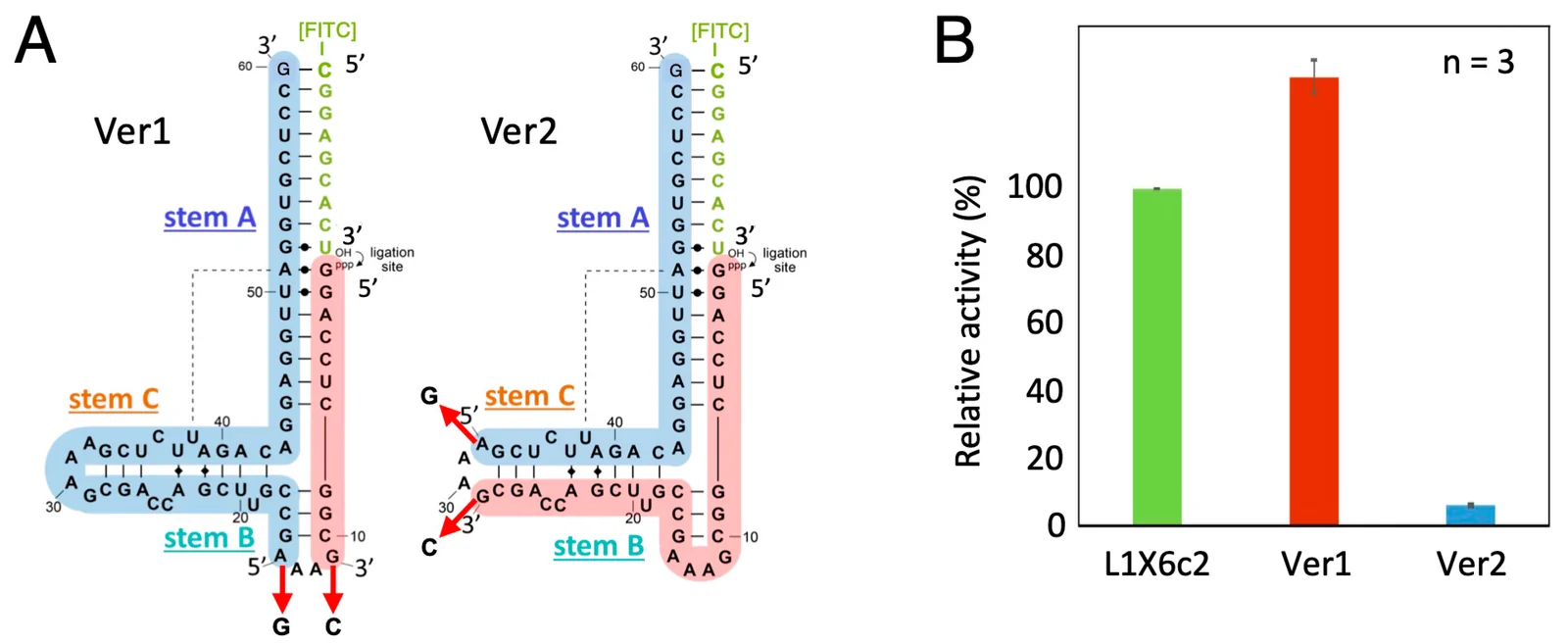
(**A**) Split mutants (versions 1 and 2) of the L1X6c2 ligase ribozyme. Red arrows indicate nucleotides that were substituted. The RNA sequences were also shown in the Supplementary Materials (Table S1). (**B**) Relative ligation-product signals of the L1X6c2 ligase ribozyme and its split mutants. The signals are shown as relative values (%) compared to that of the L1X6c2 ligase ribozyme (100%). Data are presented as mean ± SD from three independent experiments (*n* = 3).

Based on the above-mentioned results, we prepared split versions of L1X6c2 (Figure 5A). A split mutant (version 1) was designed to be cleaved at stem B by mixing an 11-nt RNA fragment (G1–G11) with a 47-nt RNA fragment (A14–G60; Figure 5A). Notably, it showed a ligation-product signal after 24 h close to that of L1X6c2 (Figure 5B). Another split mutant (version 2) was designed to be cleaved at stem C by mixing a 29-nt RNA fragment (G1–G29) with a 29-nt RNA fragment (A32–G60; Figure 5A). The relative ligation-product signal after 24 h was decreased (Figure 5B). Importantly, we introduced the G11C and A14G mutations in the former mutant (version 1) and the G29C and A32G mutations in the latter mutant (version 2; Figure 5A) based on a previous report that the transcription efficiency of T7 RNA polymerase varies significantly depending on the first base of the target RNA sequence [28,29,30].

**Figure 6:**
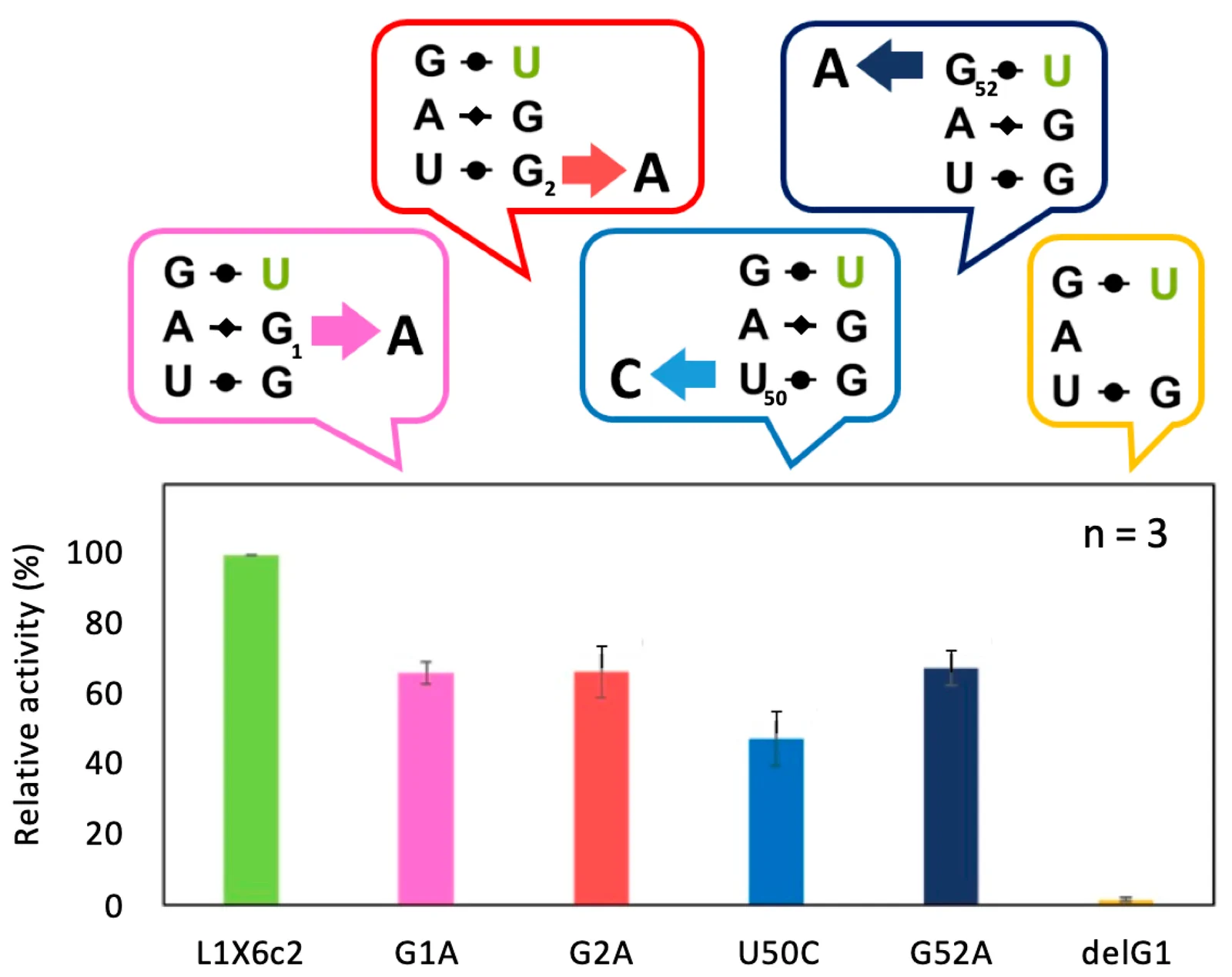
Relative ligation-product signals after 24 h of the L1X6c2 ligase ribozyme and its deletion mutants focused on the regions near the ligation site. The RNA sequences are also shown in the Supplementary Materials (Table S1). The signals are shown as relative values (%) compared to that of the L1X6c2 ligase ribozyme (100%). Data are presented as mean ± SD from three independent experiments (*n* = 3).

As bases capable of forming non-Watson–Crick base pairs were concentrated near the substrate-binding site, we generated the mutants G2A, U50C, and G52A, in which the corresponding bases were replaced with those capable of forming Watson–Crick base pairs, to determine the significance of these base pairs (Figure 6). Additionally, we generated the mutants G1A and delG1, which lack G1, and evaluated their relative ligation-product signals after 24 h. Notably, the presence of G1, but not the formation of non-Watson–Crick base pairs around the substrate-binding site, was important for ligation (Figure 6).

**Figure 7:**
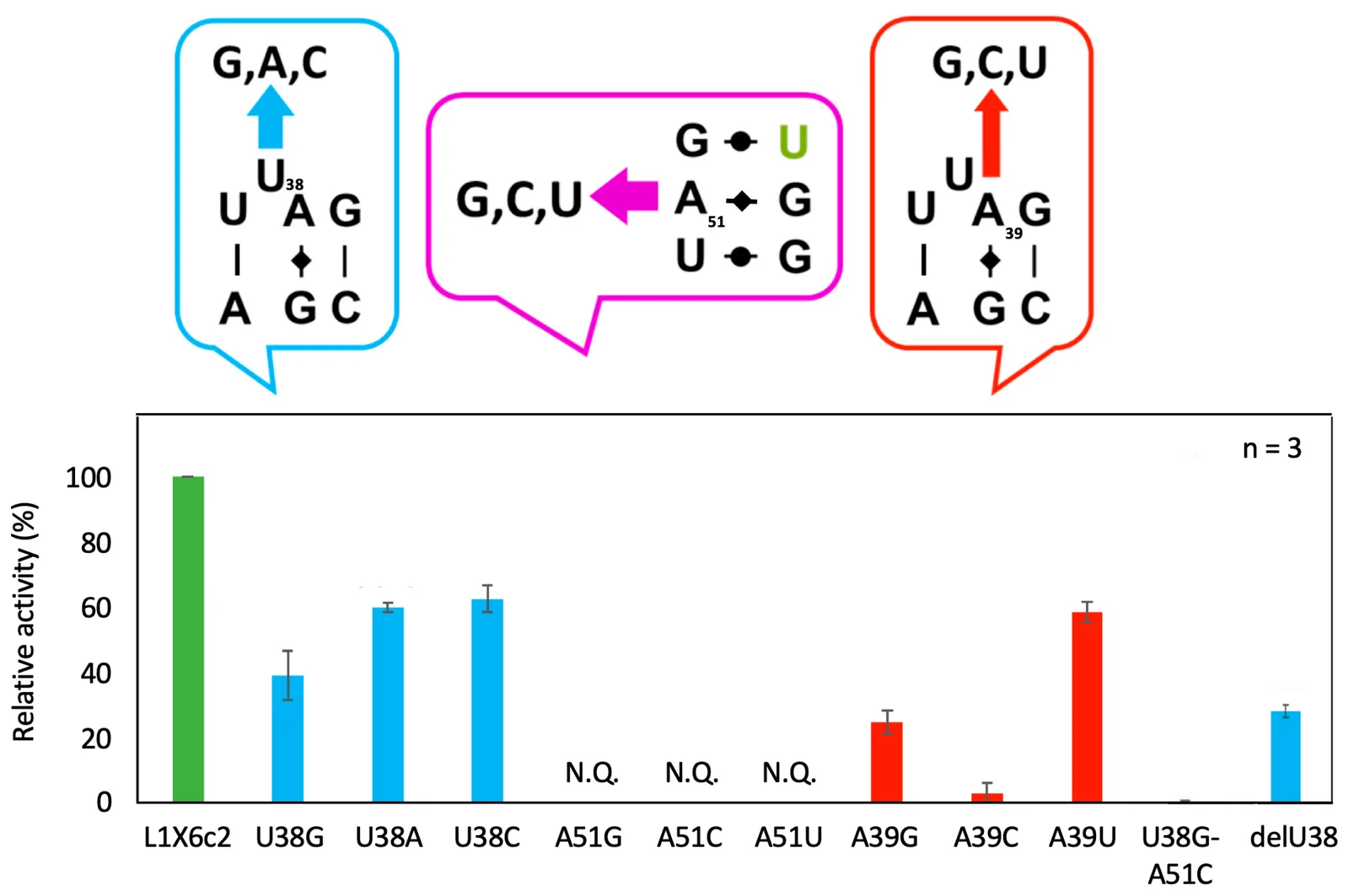
Relative ligation-product signals of the L1X6c2 ligase ribozyme and its deletion mutants focused on the regions related to a base triple and Mg^2+^ coordination. The RNA sequences are also shown in the Supplementary Materials (Table S1). The ligation-product signals are shown as relative values (%) compared to that of the L1X6c2 ligase ribozyme (100%). A signal was classified as N.Q. when the corresponding band could not be reliably quantifiable in any of the three independent PAGE analyses. Data are presented as mean ± SD from three independent experiments (*n* = 3).

As the G1–A51–U38 base triple interacted via the hydrogen bond network, we constructed the mutants U38G, U38A, U38C, A51G, A51C, and A51U by substituting U38 and A51 with their respective nucleotides (Figure 7). Furthermore, as Mg^2+^ was coordinated by G1, U38, and A39, we constructed the mutants A39G, A39C, and A39U by substituting A39 with each of the other nucleotides and evaluated their relative ligation-product signals after 24 h (Figure 7). The signal of the A39C mutant was decreased compared to those of the mutants in which A51 was substituted with other nucleotides. Next, focusing on the hydrogen bond network formed by G1–A51–U38, we generated the U38G–A51C mutant, expecting that a Watson–Crick G–C base pair would form between G38 and C51, analogous to the U38–A51 base pair, and evaluated its ligation. The U38G–A51C mutant showed markedly reduced ligation-product signal (Figure 7). To determine the mechanism by which G1–A51–U38 contributes to ligation, we generated the delU38 mutant, in which U38 was deleted, and compared its relative ligation-product signal. delU38 exhibited approximately 30% ligation-product signal (Figure 7).

## 3. Discussion

Eigen’s hypercycle theory suggests a replication limit of approximately 100 nucleotides without error correction [31,32,33]. Similarly, clay-catalyzed RNA synthesis on primitive Earth has been suggested to be restricted to <50 nucleotides [34]. The L-shaped tRNA may have evolved via the addition of an anticodon-containing arm to a pre-existing aminoacylated minihelix [35,36,37,38]. In the ribosomal peptidyl transferase center (PTC), a small RNA fragment functions as a scaffold for primordial aminoacyl-tRNAs, facilitating peptide bond formation [39,40]. Therefore, understanding the mechanisms by which short fragments of the L1 ligase ribozyme acquire catalytic activity may provide clues regarding the emergence of functional RNAs during early evolution.

It is important to note that the ligation assay used in this study was designed to compare the relative amount of ligation-product formed after 24 h under standardized conditions. Thus, the measurements represent endpoint ligation-product signals and do not provide direct information on the reaction rates or kinetic parameters of the individual variants. In particular, similar or different ligation-product signals at 24 h cannot by themselves be interpreted as evidence for similar or different catalytic efficiencies. A systematic time-course analysis of each variant would be required to determine kinetic parameters such as *k*_obs_ and the maximum ligated fraction. Such kinetic characterization was beyond the scope of the present study.

In this study, enzyme activity was lost when base pairs were simultaneously deleted from stems B and C (Δ1Δ2; Figure 3). Through activity assessment of Δ1Δ3 (51 nt), which lacks 9 nt that are considered not to be involved in the ligation reaction based on its three-dimensional structure, we successfully constructed a deletion mutant that exhibited 37% of the ligation-product signal after 24 h of L1X6c2 (Figure 4). Although stem B of Δ1Δ3 adopts a closed GAAA tetraloop structure [41] similar to that of L1X6c2, it is considered to lack sufficient length as a stem, whereas stem C is considered to adopt a structure with sufficient length by forming base pairs. A comparison of Δ2 and Δ1Δ3 confirmed that they exhibited similar levels of ligation-product signal after 24 h (Figure 3 and Figure 4). A comparison of Δ1Δ2 and Δ2 indicated that activity was lost when the stem portions of both stems B and C were simultaneously deleted, whereas activity was maintained when only the stem portion of stem B was deleted (Figure 3). This finding suggests that the stem portion of stem B is not directly involved in ligation and that the tertiary structure created by stems A and C is maintained even when the loop portion of stem B is deleted.

A ligation-product signal assessment of the deletion mutant Δ1Δ3Δ4, in which additional bases were deleted from Δ1Δ3, revealed that the signal was lost when the stem portions of both stems B and C were simultaneously deleted (Figure 4). Deletion of the internal loop further rigidifies stem C, thereby reducing its conformational flexibility. This structural change may hinder the interaction between U38 and A51, potentially resulting in the loss of activity. Subsequently, we constructed the mutants Δ1Δ3Δ5, in which two base pairs were deleted from stem A, and Δ1Δ3Δ6, in which one base pair was deleted from stem A. Notably, Δ1Δ3Δ5 (47 nt) lost its ligation-product signal; however, Δ1Δ3Δ6 (49 nt) retained its activity, showing 88% of the original ligation-product signal after 24 h (Figure 4). Furthermore, the endpoint ligation-product signal of Δ1Δ3Δ6 (49 nt) was higher than that of Δ1Δ3 (51 nt). Δ5 involves a two-base-pair deletion within a region that forms a double helix. Compared with L1X6c2, this deletion is expected to make the distance between the hinge region (near stem B) and A51 too short, while also substantially altering the orientation of the bases. In contrast, Δ6 involves only a one-base-pair deletion, and therefore its structural impact is expected to be relatively smaller than that of Δ5. In addition, the structure generated by Δ6 may be able to accommodate, to some extent, the structural alteration of Stem C caused by Δ3. We therefore speculate that the activity was restored in the Δ1Δ3Δ6 construct. In Δ3, the GCGC region is expected to be unable to form base pairs and instead adopt a looped conformation. The adjacent A-U base pair may also become destabilized. However, this structural change is not expected to substantially affect the structure on the stem A/stem B side, unlike the changes in the Δ4 region, and the flexibility of stem C may be retained to some extent.

Split variants were generated, and their relative ligation-product signals after 24 h were evaluated. Version 1, formed by cleaving L1X6c2 at stem B (Figure 5A), exhibited a higher ligation-product signal after 24 h than L1X6c2 (Figure 5B), whereas version 2, formed by cleaving L1X6c2 at stem C (Figure 5A), showed a decrease in ligation-product signal (Figure 5B). Cleavage of the loop region of stem C is expected to relieve the structural strain within the loop. Furthermore, the formation of a new C-G base pair may stabilize stem C, resulting in a more rigid structure. Consequently, the reduced flexibility of stem C may make it more difficult for U38 and A51 to interact appropriately, which may account for the decrease in activity.

**Figure 8:**
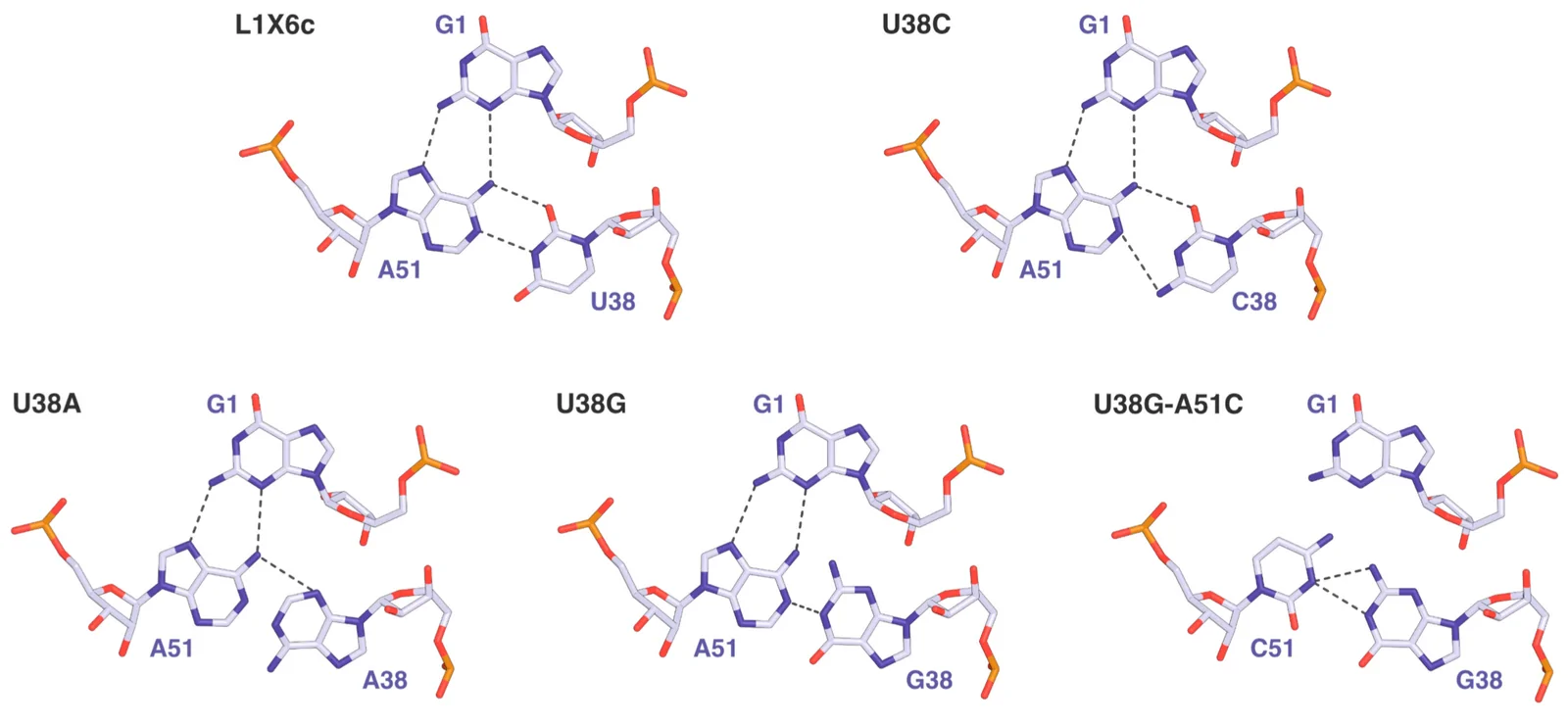
Base-triple conformations in the L1X6c ribozyme (PDB ID: 2OIU) and putative conformations of its mutants. The mutant models were generated by base substitution followed by energy minimization, as described in the Section 4. Dashed lines indicate hydrogen bonds.

Next, relative ligation-product signals of the mutants U38G, U38A, U38C, A51G, A51C, and A51U were compared. Although no base specificity was observed for U38, strong base specificity was observed for A51 (Figure 7). When the U38G–A51C mutant was generated such that the 51st and 38th bases formed a base pair, ligation-product signal was lost (Figure 7), suggesting that the presence of A51 is extremely important for ligation activity and that the G1–A51–U38 base triple contributes to the formation of the proper three-dimensional structure. Moreover, U38G–A51C did not undergo ligation under the conditions tested, whereas delU38 retained approximately 28% ligation-product signal after 24 h (lower than those of the other mutants; Figure 7), suggesting that the A51–U38 bond is relatively weak in the G1–A51–U38 base triple. Based on the base-triple conformation in the L1X6c ribozyme (PDB ID: 2OIU), mutant models were generated by base substitution (Figure 8). Because substitution of a purine with a pyrimidine caused structural clashes, each model was relaxed by restrained energy minimization using the AMBER OL3 RNA force field [42]. The hydrogen bonds between G1 and A51 are likely to be critical (Figure 8).

Comparison of the activities of the A39G, A39C, and A39U variants, in which A39 was replaced with the respective nucleotides, revealed that only the A39C variant exhibited decreased activity. This was possibly due to its inability to form a proper three-dimensional structure as G22 forms a Watson–Crick base pair with C39, resulting in reduced activity.

**Figure 9:**
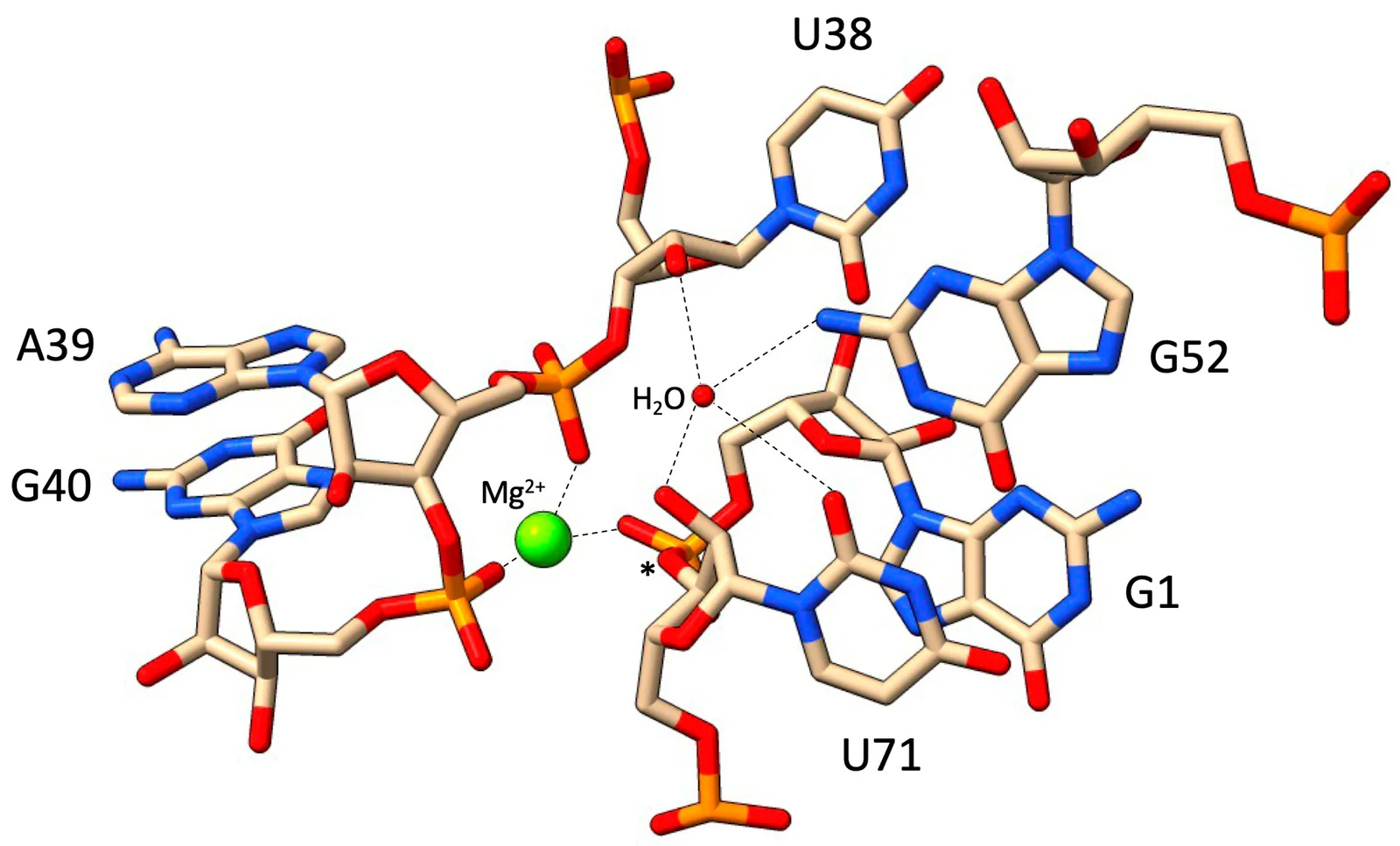
Close-up view of important interactions at the ligation site based on the tertiary structure of L1X6c (PDB ID: 2OIU). Hydrogen bonds to a water molecule and direct coordination to Mg^2+^ are indicated by black dotted lines. The 3′-oxygen atom of the 3′-terminal U at the ligation site of the substrate (corresponding to U71) is indicated by an asterisk.

Previous mutation and footprinting data [27] have suggested that the L1 ligase folds into a triple-stemmed secondary structure. The catalytic core region of approximately 17 nucleotides is located adjacent to the three-helix junction [11]. A model of a possible transition-state extrapolated from observed atomic positions in L1X6c has suggested that the O1P phosphate of A39 is ideally positioned to abstract a proton from the 3′-oxygen of the 3′-terminal U of the substrate (corresponding to U71), thus generating an attacking nucleophile (Figure 9) [11]. Based on the structure of L1X6c, the 2′-hydroxyl of the 3′-terminal U of the substrate has a tightly bound water molecule as a hydrogen-bonding partner. This water molecule interacts specifically not only with the 2′-hydroxyl of 3′-terminal U of the substrate but also with the 2′-hydroxyl of U38, N2 amino group of G52, and O2 oxygen of 3′-terminal U of the substrate. This hydrogen-bonding network may keep the 2′-hydroxyl of U away from the ligation-site phosphate. If deprotonation occurs, 2′-alkoxide may be re-protonated by the water molecule, thereby suppressing the side reaction (Figure 9) [11].

The L1 ligase ribozyme folds into a compact structure in which a set of invariant nucleotides interacts to create a catalytic pocket capable of juxtaposing the ligation ends with a bound Mg^2+^ ion cofactor [11]. Unexpectedly, the activity was almost maintained even when non-Watson–Crick base pairs near the ligation site were changed to Watson–Crick base pairs. Although the activity was reduced in delG1, the presence of G1 was important for ligation (Figure 7).

Transition-state stabilization interactions and functional group positioning for general base catalysis are crucial for promoting phosphodiester bond formation. Among in vitro evolved ligase ribozymes, the L1 and R3C ligases show lower catalytic rates (0.1/min) [42] than the class I ligase ribozyme (100/min) [43], whose catalytic rate is close to that of natural proteinaceous polymerases. In contrast, the transition-state stabilization models built for the class I ligase [23,44] and proteinaceous polymerases [42,45] are similar; such conformational arrangements are not observed in the L1 ligase [11]. These differences are possibly responsible for the lower catalytic rate of the L1 ligase.

Although the short length of the 49 nt ligase is intriguing in the context of the RNA world hypothesis, caution is warranted in extending our findings to prebiotic RNA evolution. The ribozyme examined here is an experimentally designed variant and was characterized under defined laboratory conditions rather than under experimentally demonstrated prebiotic conditions. Therefore, this study should not be taken as direct evidence for the origin or evolutionary history of catalytic RNA on the early Earth. Instead, our results establish a laboratory model for exploring the extent to which catalytic function can be retained in highly truncated RNA sequences. Such a model may help inform future studies aimed at understanding the constraints and possibilities associated with short functional RNAs in prebiotic and early evolutionary contexts.

## 4. Materials and Methods

### 4.1. Preparation of L1 Ligase Ribozyme Variants and RNA Substrate

Unlabeled deoxyribonucleotides were synthesized by Eurofins Genomics K.K. (Tokyo, Japan). Each template DNA was prepared from chemically synthesized deoxyribonucleotides carrying the T7 promoter and the sequences corresponding to variants of the L1X6c2 ligase ribozyme, and two synthetic primers were prepared using the polymerase chain reaction. RNA transcription was performed at 37 °C for 16 h in a reaction mixture containing 40 mM Tris-HCl (pH 8.0), 10 mM dithiothreitol, 2 mM spermidine, 8 mM MgCl_2_, 2.5 mM each NTP, template DNA (0.2 mg/mL), and T7 RNA polymerase (~100 μg/mL) partially purified using Ni-NTA agarose chromatography [46,47]. The transcripts were purified by denaturing 12% polyacrylamide gel electrophoresis. The concentrations of purified obtained RNA were determined from the UV absorbance at a wavelength of 260 nm using an Implen NanoPhotometer (Implen GmbH, München, Germany). The HPLC-purified 5′-terminal fluorescein-5-isothiocyanate (FITC)-labeled oligonucleotide (5′-FITC-CGGAGCACU-3′) was prepared by Fasmac Co., Ltd. (Atsugi, Japan) and was used as an RNA substrate in the ligation reaction. All of the RNA sequences of ribozymes used in this study are shown in the Supplementary Materials (Table S1).

### 4.2. Analysis of Ligation

Ligation analysis was performed by the method of Rogers and Joyce with a slight modification [17]. The L1X6c2 ligase ribozyme variants, dissolved in solution containing 50 mM Tris-HCl (pH 8.5) and 15 mM MgCl_2_ were first heated to 37 °C for 5 min and then cooled to 4 °C. Then, the ligation reaction was started by adding 1.5 µL of 50 µM substrate to the solution. The final concentration of the ribozyme and the substrate were 1 µM and 5 µM, respectively. The volume of the reaction mixture was 15 µL. After incubation at 23 °C for 24 h, the solution was applied to denaturing 12% polyacrylamide gel containing 7 M urea for electrophoresis. The gel was analyzed on a Typhoon FLA 7000 (GE Healthcare Japan, Tokyo, Japan), and the ligated products were quantified by using ImageQuant TL software (Version 7.0).

Ligation reactions were performed in 50 mM Tris-HCl (pH 8.5) containing 15 mM MgCl_2_. The pH 8.5 condition was selected based on preliminary comparisons showing that, although pH 9.0 gave a higher initial ligation signal, the signal decreased substantially by 24 h, whereas it remained high at pH 8.5.

Following electrophoresis, ligation-product bands were assigned according to their expected migration positions relative to the fluorescently labeled substrate, the unligated ribozyme, and the tracking dyes xylene cyanol FF and bromophenol blue. Background fluorescence was subtracted from the measured band intensity. Fluorescence images were acquired under identical imaging conditions for samples analyzed within each experiment. The regions corresponding to the substrate and ligation-product bands were used for fluorescence quantification. For each gel, identical-sized rectangular regions of interest were used to quantify the fluorescence intensity of the ligated product generated by L1X6c2, the ligated product generated by each mutant ribozyme, and a gel region without loaded sample to determine the local gel background. The background-corrected fluorescence intensity was calculated by subtracting the fluorescence intensity of the background region from that of the corresponding ligation band. A gel image from a representative independent experiment is provided in the Supplementary Materials (Figure S1) to document the band assignment procedure used for the quantitative analysis. “N.Q.” indicates that the ligation-product signal could not be reliably quantified under the conditions used.

### 4.3. Molecular Modeling

All models were generated from chain Q of the L1X6c crystal structure (PDB ID: 2OIU), and mutations were introduced with the Mutagenesis Wizard in PyMOL 3.1.0 (Schrödinger, LLC., New York, NY, USA). Systems were prepared using the tleap module of AmberTools 26 [48] with the ff99bsc0χ_OL3_ force field for RNA [49] and the Li–Merz 12-6 parameters for Mg^2+^ [50]. Each system was solvated in a truncated octahedral box of TIP3P water [51] with a 12 Å buffer, neutralized with Na^+^, and adjusted to 150 mM NaCl. Energy minimization was performed with pmemd in Amber 26 [52] using 2500 steps of steepest descent followed by 2500 steps of conjugate gradient minimization with a 10 Å nonbonded cutoff. Harmonic positional restraints of 10 kcal mol^−1^ Å^−2^ were applied to all RNA heavy atoms and crystallographic Mg^2+^ ions. Hydrogen bonds were identified using the find polar contacts function in PyMOL.

## 5. Conclusions

In this study, evaluation using split variants revealed that the formation of a proper tertiary structure by stems A and C was responsible for ligation. The Δ1Δ3Δ6 construct (49 nt), which lacked 11 nt including two bases in stem A, the stem portion of stem B, and the loop portion of stem C, exhibited 88% of the activity of L1X6c2, indicating the successful development of a ligase ribozyme with sufficient activity at ≤50 nt. The split mutant with a break in stem C strongly reduced the ligation-product signal compared to that of L1X6c2, suggesting that two distinct RNA strands adopt a proper tertiary structure conducive to ligation. The actual structure of Δ1Δ3Δ6, which may adopt an ideal conformation owing to some form of interaction, remains unknown, warranting further investigation. Furthermore, our findings suggest that the G1–A51–U38 base triple is crucial for the formation of this three-dimensional structure and that interactions between G1 and A51, in particular, are important for the strong base specificity of A51.

In conclusion, our study provides experimental evidence based on measurements of the ligation-product signal and identifies sequence features. We note that these endpoint measurements alone do not allow us to directly determine the ribozyme’s tertiary structure or to establish that a particular stem adopts a specific conformation. Thus, the structural models and assignments discussed here are best considered hypotheses that provide a possible interpretation of the observed activity, rather than definitive structural conclusions. Direct structural or biophysical measurements will be necessary to distinguish among these possibilities and to establish the relationship between the proposed structural features and ribozyme function.

## Data Availability

The data presented in this study are available from the corresponding authors upon reasonable request.

## Supplementary Materials

The following supporting information can be downloaded at https://www.mdpi.com/article/10.3390/ijms27209107/s1.
