# Supplementary material for: Analysis of Ligation Activity in Miniaturized L1 Ligase Ribozyme Variants Down to 49 nt and in Split Ribozyme Forms
Source: Int J Mol Sci. 2026 Oct 14;27(20):9107. doi: 10.3390/ijms27209107 (PMC13705289; doi:10.3390/ijms27209107)
Supplement: Supplementary file 1 [file ijms-27-09107-s001.zip › ijms-4474257-supplementary.pdf]

Supplementary Materials for

Analysis of Ligation Activity in Miniaturized L1 Ligase Ribozyme Variants Down to 49 nt and in Split Ribozyme Forms

Yuki Nitta[1], Misaki Kato[1], Yunoka Takagi[1], Yusuke Saga[1], Tadashi Ando[2,3], Hiromi Mutsuro-Aoki[1], and Koji Tamura[1,3,*]

[1]Department of Biological Science and Technology, Tokyo University of Science, 6-3-1 Niijuku, Katsushika-ku, Tokyo 125-8585, Japan

[2]Department of Applied Electronics, Tokyo University of Science, 6-3-1 Niijuku, Katsushika-ku, Tokyo 125-8585, Japan

[3]Research Institute for Science and Technology, Tokyo University of Science, 2641 Yamazaki, Noda 278-8510, Japan

**Correspondence. Email: koji@rs.tus.ac.jp*

This PDF file includes:

Figure S1

Table S1

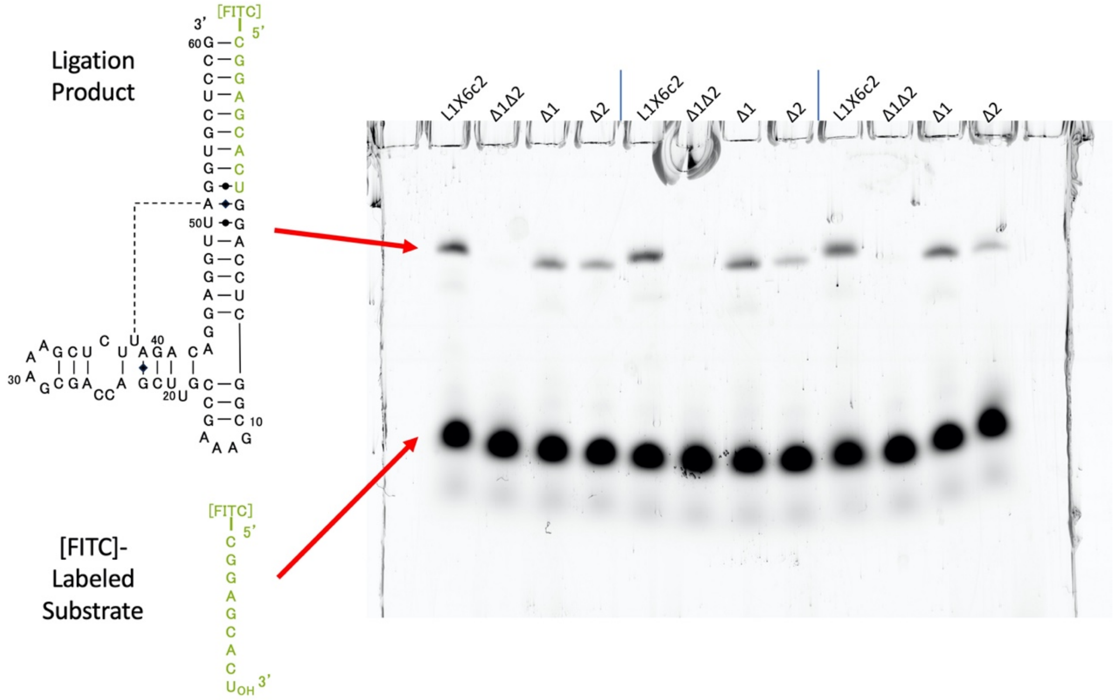

Figure S1. Gel image corresponding to Figure 3. The positions of the fluorescently labeled substrate and ligation-product are indicated. The gel was visualized using a Typhoon FLA 7000 (GE Healthcare Japan, Tokyo, Japan), and the ligation-product band was quantified from the gel image using ImageQuant TL software, as described in the Materials and Methods. The same electrophoretic and fluorescence-based analyses were performed for all corresponding experiments.

Table S1. RNA sequences of the ribozymes used in this study.

RNA	Sequence (5′ to 3′)	Length
L1X6c2	GGACCUCGGCGAAAGCCGUUCGACCAGCGAAAGCUCUUAGACAGGAGGUUAGGUGCUCCG	60 nt
Δ1Δ2	GGACCUCGGAAACGUUCGAGCGAAAGCUUAGACAGGAGGUUAGGUGCUCCG	51 nt
Δ1	GGACCUCGGAAACGUUCGACCAGCGAAAGCUCUUAGACAGGAGGUUAGGUGCUCCG	56 nt
Δ2	GGACCUCGGCGAAAGCCGUUCGAGCGAAAGCUUAGACAGGAGGUUAGGUGCUCCG	55 nt
Δ1Δ3	GGACCUCGGAAACGUCGACCAGCGCUCUUAGACAGGAGGUUAGGUGCUCCG	51 nt
Δ1Δ3Δ4	GGACCUCGGAAACGUCGAAGCGCUUUAGACAGGAGGUUAGGUGCUCCG	48 nt
Δ1Δ3Δ5	GGACCGGAAACGUCGACCAGCGCUCUUAGACAGGGUUAGGUGCUCCG	47 nt
Δ1Δ3Δ6	GGACCUGGAAACGUCGACCAGCGCUCUUAGACAGAGGUUAGGUGCUCCG	49 nt
Version 1	GGACCUCGGCC + GGCCGUUCGACCAGCGAAAGCUCUUAGACAGGAGGUUAGGUGCUCCG	11+47 nt
Version 2	GGACCUCGGCGAAAGCCGUUCGAGCC + GGCUCUUAGACAGGAGGUUAGGUGCUCCG	26+29 nt
G1A	AGACCUCGGCGAAAGCCGUUCGACCAGCGAAAGCUCUUAGACAGGAGGUUAGGUGCUCCG	60 nt
G2A	GAACCUCGGCGAAAGCCGUUCGACCAGCGAAAGCUCUUAGACAGGAGGUUAGGUGCUCCG	60 nt
U50C	GGACCUCGGCGAAAGCCGUUCGACCAGCGAAAGCUCUUAGACAGGAGGUCAGGUGCUCCG	60 nt
G52A	GGACCUCGGCGAAAGCCGUUCGACCAGCGAAAGCUCUUAGACAGGAGGUUAAGUGCUCCG	60 nt
delG1	GACCUCGGCGAAAGCCGUUCGACCAGCGAAAGCUCUUAGACAGGAGGUUAGGUGCUCCG	59 nt
U38G	GGACCUCGGCGAAAGCCGUUCGACCAGCGAAAGCUCUGAGACAGGAGGUUAGGUGCUCCG	60 nt
U38A	GGACCUCGGCGAAAGCCGUUCGACCAGCGAAAGCUCUAAGACAGGAGGUUAGGUGCUCCG	60 nt
U38C	GGACCUCGGCGAAAGCCGUUCGACCAGCGAAAGCUCUCAGACAGGAGGUUAGGUGCUCCG	60 nt
A51G	GGACCUCGGCGAAAGCCGUUCGACCAGCGAAAGCUCUUAGACAGGAGGUUGGGUGCUCCG	60 nt
A51C	GGACCUCGGCGAAAGCCGUUCGACCAGCGAAAGCUCUUAGACAGGAGGUUCGGUGCUCCG	60 nt
A51U	GGACCUCGGCGAAAGCCGUUCGACCAGCGAAAGCUCUUAGACAGGAGGUUUGGUGCUCCG	60 nt
A39G	GGACCUCGGCGAAAGCCGUUCGACCAGCGAAAGCUCUUGGACAGGAGGUUAGGUGCUCCG	60 nt
A39C	GGACCUCGGCGAAAGCCGUUCGACCAGCGAAAGCUCUUCGACAGGAGGUUAGGUGCUCCG	60 nt
A39U	GGACCUCGGCGAAAGCCGUUCGACCAGCGAAAGCUCUUUGACAGGAGGUUAGGUGCUCCG	60 nt
U38G-A51C	GGACCUCGGCGAAAGCCGUUCGACCAGCGAAAGCUCUGAGACAGGAGGUUCGGUGCUCCG	60 nt
delU38	GGACCUCGGCGAAAGCCGUUCGACCAGCGAAAGCUCUAGACAGGAGGUUAGGUGCUCCG	59 nt
